# Gut microbiota regulation and Traditional Chinese Medicine syndrome differentiation in right- and left-sided colon cancer heterogeneity — A review

**DOI:** 10.3389/fphar.2026.1768252

**Published:** 2026-06-12

**Authors:** Jiatian Chen, Jinting Zhao, Xiaoxiao Zhang, Huirong Zhu

**Affiliations:** 1 Putuo Hospital, Shanghai University of Traditional Chinese Medicine, Shanghai, China; 2 The First School of Clinical Medicine, Zhejiang Chinese Medical University, Hangzhou, China; 3 Shanghai University of Traditional Chinese Medicine, Shanghai, China

**Keywords:** gut microbiota, left-sided colon cancer, right-sided colon cancer, spatial heterogeneity, syndrome differentiation, Traditional Chinese Medicine (TCM)

## Abstract

Colon cancer (CC) exhibits substantial heterogeneity between right-sided colon cancer (RCC) and left-sided colon cancer (LCC) in terms of embryological origin, molecular characteristics, immune microenvironment, and therapeutic response. Increasing evidence suggests that spatial heterogeneity of the gut microbiota may contribute to these site-specific differences through interactions with local immune, inflammatory, and metabolic microenvironments. Recent studies have also indicated that gut microbiota dysbiosis may participate in the progression, therapeutic response, and microenvironmental remodeling of CC. This review summarizes current evidence regarding the role of gut microbiota spatial heterogeneity in RCC and LCC, with particular emphasis on its potential associations with molecular features, immune regulation, and local metabolic alterations. Furthermore, we discuss recent advances in Traditional Chinese Medicine (TCM)-based interventions targeting the gut microbiota in CC, including potential links between common TCM syndromes, microbial dysbiosis, mucosal barrier dysfunction, and immune-inflammatory alterations. We also summarize the regulatory effects of representative botanical monomers and classical TCM formulas on microbial homeostasis and tumor-related microenvironmental changes. Importantly, this review does not aim to establish fixed correspondences between specific TCM syndromes and tumor location. Instead, gut microbiota heterogeneity may serve as a modern biological reference for understanding site-specific pathogenesis and guiding individualized TCM interventions in CC.

## 1 Introduction

Colorectal cancer (CRC) is a malignant tumor originating from the epithelial tissues of the colon or rectum. According to 2022 global cancer statistics, approximately 1.926 million new CRC cases and 904,000 CRC-related deaths occur worldwide each year, accounting for 9.6% of all newly diagnosed cancers and 9.3% of all cancer-related deaths, respectively (Bray et al., 2024). As a major subtype of CRC, CC is clinically characterized by changes in bowel habits, abdominal pain, abdominal masses, and unexplained anemia, and contributes significantly to the overall disease burden of CRC (McDermott et al., 1981; Ford et al., 2008; Argilés et al., 2020). In recent years, CC has shown a trend toward earlier onset, and tumors arising from different anatomical sites exhibit differences in epidemiology, pathological characteristics, therapeutic response, and prognosis, further highlighting the necessity of investigating CC heterogeneity to guide precision prevention and treatment strategies (Liu et al., 2025).

Clinical studies have shown that RCC and LCC exhibit marked heterogeneity in terms of embryological origin, molecular features, immune microenvironment, and therapeutic response. The right colon primarily originates from the midgut and is characterized by water absorption and anaerobe-dominated fermentative metabolism, whereas the left colon originates from the hindgut and is more involved in fecal storage, propulsion, and excretion. These anatomical and physiological differences collectively shape the spatial heterogeneity of the gut microbiota across different colonic sites and may further influence the local metabolic environment and mucosal immune status, thereby contributing to site-specific tumor biology in CC.

In recent years, the role of the gut microbiota in the development and progression of CC has attracted increasing attention. Studies have shown that spatial differences in microbial distribution exist across different colonic sites even under healthy conditions, whereas CC patients often present with dysbiosis, characterized by a reduction in beneficial bacteria and an increased relative abundance of opportunistic pathogens (Taniguchi et al., 2016; Kosumi et al., 2018; Asadollahi et al., 2020). The gut microbiota may participate in tumorigenesis and tumor progression by producing metabolites, regulating inflammatory responses, and modulating the immune microenvironment. However, the causal relationship between microbial spatial heterogeneity and the biological differences between RCC and LCC remains to be further investigated.

With the continued advancement of modern oncology and TCM research, the role of TCM in the comprehensive prevention and treatment of CC has gained increasing recognition. TCM syndrome differentiation emphasizes the overall condition, individual differences, and dynamic evolution of pathogenesis, rather than classification based solely on anatomical location. Given the differences in microbial niches and local microenvironments between RCC and LCC, the gut microbiota may play an important role in the pathogenesis of CC at different anatomical sites. Modern studies have shown that active botanical metabolite s and formulas may not only improve clinical symptoms and reduce radiochemotherapy-related adverse effects, but also participate in the regulation of the tumor microenvironment by modulating gut microbiota composition, enhancing mucosal barrier function, and influencing local immune-inflammatory status (Shen et al., 2010; Kostic et al., 2012; Louis et al., 2014; Repass et al., 2016; Chen et al., 2020). Therefore, the gut microbiota may provide a new perspective for understanding site-specific pathogenesis in CC and for guiding individualized TCM interventions.

Therefore, this review systematically summarizes the differences between RCC and LCC in terms of anatomical structure, molecular features, immune environment, and spatial distribution of the gut microbiota, with a focus on the potential links between gut microbiota-mediated local microenvironmental changes and the pathogenic features of CC. Furthermore, this review summarizes recent advances in TCM interventions targeting the gut microbiota in CC, aiming to provide a theoretical reference for individualized TCM interventions based on microbial heterogeneity.

## 2 Literature search strategy

Relevant literature published up to January 2026 was retrieved from PubMed, Web of Science, CNKI, and Google Scholar databases. The search strategy combined keywords related to colorectal cancer/colon cancer heterogeneity, gut microbiota, and Traditional Chinese Medicine (TCM), including “colorectal cancer,” “CRC,” “colon cancer,” “CC,” “right-sided colon cancer,” “left-sided colon cancer,” “right-sided colorectal cancer,” “left-sided colorectal cancer,” “gut microbiota,” “microbiome,” “Traditional Chinese Medicine,” “TCM syndrome,” “herbal medicine,” “tumor microenvironment,” and “microbial metabolites.” Boolean operators such as “AND” and “OR” were used when appropriate.

Both experimental and clinical studies investigating the relationships among gut microbiota, CRC/CC heterogeneity, immune regulation, metabolic alterations, and TCM interventions were included. Because many studies on microbiota, TCM interventions, and tumor microenvironmental regulation are conducted in CRC rather than CC-specific cohorts, CRC-related studies were also considered when their findings were relevant to colon cancer biology or could provide mechanistic insights into CC. Priority was given to studies with relatively clear mechanistic evidence, representative clinical findings, or relevance to microbiota-related regulation in CC. Additional relevant studies were identified through manual screening of reference lists from selected publications.

Duplicate publications, articles lacking relevance to the topic, conference abstracts without sufficient data, and studies without accessible full texts were excluded. Given the narrative nature of this review, the included evidence was interpreted qualitatively rather than through formal meta-analysis or systematic review methodology.

## 3 Biological differences between RCC and LCC

### 3.1 Anatomical and physiological differences and spatial distribution of gut microbiota

The right and left colon differ significantly in terms of embryological origin, blood supply, luminal function, and microbiota composition (Table 1). These differences collectively shape distinct gut microbial niches and provide an important biological basis for the heterogeneity between RCC and LCC in molecular features, immune microenvironment, metabolic status, and clinical manifestations (Yamashita et al., 2018). In general, the right colon primarily originates from the midgut and is characterized by water and electrolyte absorption as well as anaerobic fermentation. It contains relatively abundant fermentable carbohydrate substrates, creating a more acidic local environment and supporting the enrichment of anaerobic fermentation-related bacteria (Sommer and Bäckhed, 2013). As intestinal contents move toward the left colon, water is continuously reabsorbed, carbohydrate substrates gradually decrease, and protein fermentation becomes relatively enhanced. Consequently, the luminal pH shifts toward neutral or slightly alkaline conditions. Meanwhile, the mucus layer and barrier structure also undergo changes, gradually transitioning from a loosely organized structure to a dense bilayer (Wu et al., 2011). Given that this review focuses on the potential links between gut microbiota heterogeneity and TCM syndrome-based interventions, this section provides only a brief overview of the basic anatomical, physiological, and microbial differences between the right and left colon, as summarized in Table 1.

**TABLE 1:** Comparison of right- and left-sided colon in terms of anatomy, physiology, and microbiota.

Category	Right-sided colon	Left-sided colon	References (PMID)
Embryological origin	Midgut	Hindgut	Bara et al. (1984)
Anatomical distribution	Cecum, ascending colon, and proximal transverse colon (60% of colon)	Distal transverse colon, descending colon, and sigmoid colon (40% of colon)	Baijal et al. (1998)
Blood supply	Superior mesenteric artery (≈60%)	Inferior mesenteric artery (≈40%)	Sommer and Bäckhed (2013)
Predominant physiological functions	Water and electrolyte absorption, Anaerobic carbohydrate fermentation	Fecal storage and propulsion	Pata et al. (2021)
pH range	5.7–6.1 (relatively acidic)	6.5–7.0 (neutral to mildly alkaline)	Louis et al. (2014), Ou et al., (2022)
Microbiota composition	Enriched anaerobic bacteria, including *Fusobacterium nucleatum* and *Desulfovibrio*	Enriched commensal bacteria, including *Prevotella*, *Roseburia*, and *Faecalibacterium prausnitzii*	Ali et al. (2026), Li and Dong (2026)
Typical clinical presentation	Occult bleeding, anemia, fatigue, abdominal pain, and bloating	Hematochezia, altered bowel habits, constipation, and intestinal obstructio	Ciardiello et al. (2022)

### 3.2 Differences in molecular genetic characteristics

RCC and LCC differ significantly in microsatellite stability, chromosomal instability, driver gene mutation profiles, and the distribution of consensus molecular subtypes (CMS) (Table 2). In general, RCC more commonly exhibits microsatellite instability-high (MSI-H) or deficient mismatch repair (dMMR) features, accompanied by a higher frequency of the BRAF V600E mutation and the CpG island methylator phenotype (CIMP) (Goldstein et al., 2014). In contrast, LCC is more prominently characterized by chromosomal instability (CIN) and activation of the WNT signaling pathway (Manne et al., 1998; Xicola et al., 2014). Regarding CMS classification, RCC is relatively enriched in the immune-activated CMS1 subtype, whereas LCC more frequently presents as CMS2, which is characterized by epithelial proliferation and activation of WNT/MYC signaling. These molecular differences not only influence the tumor biology of RCC and LCC but are also closely associated with their differential sensitivity to immunotherapy and anti-EGFR targeted therapy (Qi et al., 2025). Recent multi-cohort studies suggest that the distribution of certain driver gene mutations between RCC and LCC may be more complex than previously recognized; for example, KRAS mutation rates vary across different studies (Saravani et al., 2021). However, differences in study design, patient populations, sequencing platforms, and analytical methods may partly explain the inconsistent mutation frequencies reported across cohorts. Therefore, the molecular distinction between RCC and LCC should be regarded as a general tendency rather than a strict one-to-one relationship between tumor sidedness and specific driver gene alterations. Accordingly, RCC and LCC should not be distinguished solely by a single molecular event but should instead be understood from multiple dimensions, including anatomical site, molecular subtype, immune status, and microbial niche. Importantly, molecular genetic differences alone cannot fully explain the clinical heterogeneity between RCC and LCC; spatial microbial distribution, the local metabolic environment, and mucosal immune status may collectively contribute to this heterogeneity and provide a basis for further understanding differences in symptom presentation and therapeutic responses from the perspective of TCM syndrome differentiation.

**TABLE 2:** Molecular and genetic differences between RCC and LCC.

Category	RCC	LCC	References (PMID)
Microsatellite instability (MSI-H)	High frequency (20%–30%) Strongly associated with dMMR	Low frequency (5%–10%)	Sinicrope et al. (2006), Overman et al. (2017), Wu et al. (2023)
Chromosomal instability (CIN)	Lower frequency	Higher frequency	Christie et al. (2013)
*BRAF* V600E mutation	Detected in ∼15% (8.5%–22.5%); associated with CIMP and MSI-H; often linked to poor prognosis	Detected in ∼2–5%	Jafry et al. (2024), Abdelgadir et al. (2025)
*KRAS* mutation	Reported in ∼46%	Reported in ∼36% Previously considered more frequent in LCC	Manne et al. (1998), Xicola et al. (2014), Saravani et al. (2021)
Consensus molecular subtypes (CMS)	Predominantly CMS1 (∼30–40%); immune-active subtype	Predominantly CMS2 (∼50–60%); WNT-activated subtype	Guinney et al. (2015), Dienstmann et al. (2017)
Therapeutic response pattern	May benefit from immune checkpoint inhibitor (ICI) therapy, particularly in MSI-H/dMMR tumors	May derive greater benefit from anti-EGFR therapy in molecularly selected patients	Baran et al. (2018)

## 4 Mechanistic of the gut microbiota in CC

The gut microbiota and its metabolites play a key role in the development and progression of CC. Under healthy conditions, the gut microbiota helps maintain intestinal homeostasis by participating in nutrient metabolism, preserving intestinal barrier integrity, and regulating immune balance (Tudorache et al., 2025). When microbial homeostasis is disrupted, dysbiotic microbiota and their metabolites may contribute to tumor initiation and progression through multiple mechanisms, including the modulation of inflammatory responses, barrier function, metabolic pathways, and anti-tumor immunity (Li et al., 2026). However, much of the current evidence is still derived from mechanistic studies, animal experiments, or relatively small clinical cohorts, and differences in study populations and analytical methods may also affect the consistency of reported findings. Therefore, the causal role of specific microbiota-related mechanisms in human CC progression still requires further validation.

### 4.1 Potential associations between gut microbiota, molecular characteristics, and therapeutic differences in RCC and LCC

The microbiota at different anatomical sites of the colon may participate in shaping molecular features, immune status, and therapeutic responses (Rosty et al., 2013; James et al., 2020; Wang M. et al., 2023) (Table 3). In RCC, *F. nucleatum* can induce chronic inflammation and oxidative stress through the TLR4/NF-κB axis and may be associated with molecular features such as MLH1 methylation silencing, MSI-H, and CIMP-H (Mima et al., 2016a). H_2_S produced by Desulfovibrio is genotoxic and may cause DNA double-strand breaks, thereby exacerbating genomic instability (Yachida et al., 2019; Zeng et al., 2019). Enterotoxigenic *Bacteroides fragilis (ETBF)* can activate the STAT3 pathway, thereby promoting tumor cell proliferation and inhibiting apoptosis (Ou et al., 2022). The persistent accumulation of these bacteria in the right colon may contribute to a CMS1-like tumor microenvironment characterized by a high mutational burden and immune activation, which may partly underlie the relatively greater sensitivity of RCC patients to immune checkpoint inhibitors (ICIs) (Li et al., 2026). In contrast, certain bacteria in LCC can convert primary bile acids into deoxycholic acid (DCA), which may promote tumor progression through the MAPK/ERK and FXR/SHP pathways and may be associated with CIN and KRAS mutations (Degirolamo et al., 2011). Additionally, colibactin-producing *E. coli* can induce DNA damage and may cooperate with DCA to promote the formation of a local pro-tumor microenvironment (Pleguezuelos-Manzano et al., 2020). Meanwhile, LCC generally exhibits lower immune cell infiltration and relatively insufficient immunogenicity (Liu et al., 2024). However, the evidence supporting this interpretation remains incomplete, as some findings are derived from bioinformatic analyses and still require validation in experimental studies and independent clinical cohorts. These immune-related differences may partly explain why therapeutic strategies for LCC more often focus on specific signaling pathways, such as anti-EGFR monoclonal antibody therapy, rather than ICI-centered immunotherapy. Collectively, these findings suggest that spatial heterogeneity of the gut microbiota may contribute to the distinct molecular features and therapeutic responses observed in RCC and LCC, offering new perspectives for microbiota-based individualized interventions.

**TABLE 3:** Summary of heterogeneity and mechanistic differences in gut microbiota distribution between RCC and LCC.

Comparison dimension	RCC	LCC	References (PMID)
Dominant bacterial genera	*Fusobacterium nucleatum, Desulfovibrio* (relative abundance reported to be 2–3-fold higher than in LCC)	*Bacteroides, Roseburia, Faecalibacterium prausnitzii, Lactobacillus* (reported to be 2–3-fold higher than in RCC)	Donaldson et al. (2016), Mima et al. (2016b), Nosho et al. (2016), Chen S. et al. (2022), Cao et al. (2023)
Biofilm coverage rate	Reported biofilm coverage up to 60%	Reported biofilm coverage of ∼15–20%	Kim et al. (2018)
Representative microbial metabolites	Relatively higher levels of SCFAs and primary bile acids	Relatively enriched secondary bile acids (e.g., DCA)	van Gorkom et al. (1997), Parada Venegas et al. (2019)
Microbiota-driven carcinogenic mechanisms	DNA damage; activation of Wnt/β-catenin, STAT3, and NF-κB signaling; chronic inflammation	SCFA-mediated GPR43 activation; anti-inflammatory and anti-tumor immune effects	Qi et al. (2025)
Association with molecular features	High *F. nucleatum* abundance associated with MSI-H, *BRAF* mutation, and CIMP	SCFA-producing bacteria associated with CMS2/3 subtypes and EGFR-related signaling	Guinney et al. (2015)
Association with immune microenvironment	Associated with enrichment of M2-type TAMs and Tregs and an immunosuppressive microenvironment	Associated with increased Th17 and CD8^+^ T-cell activity	Dienstmann et al. (2017)
Tumor-associated macrophages (M2 TAMs)	Higher infiltration (∼40–45%); associated with IL-10, TGF-β, and PD-L1-mediated immunosuppression	Relatively lower infiltration	Kneis et al. (2023), Liang et al. (2024)
Regulatory T cells (Tregs)	Increased proportion; associated with enhanced immunosuppression	Lower abundance than RCC; more localized at tumor margins	Hu et al. (2025)
Helper T cells (Th17)	Lower density	Higher density (∼1.5-fold vs. RCC); associated with IL-17 and IL-2-related inflammatory responses	Guo et al. (2022)
CD8^+^ T cells	Higher infiltration but relatively lower activation; increased PD-1 expression (20%–30%)	Relatively lower infiltration and PD-1 expression (10%–15%)	Taieb et al. (2018)
Natural Killer (NK) cells	Lower number and activity	Higher abundance and IFN-γ production; stronger anti-tumor activity	Kwak and Ju (2019)
B cells	Lower density	Higher density at tumor margins; potentially involved in antibody-mediated immune responses	de Oliveira et al. (2024)

### 4.2 Differences in the immune and metabolic microenvironments between RCC and LCC

RCC often exhibits higher immune infiltration, but this may also be accompanied by the enrichment of immunosuppressive cells. *F. nucleatum* can bind to TIGIT via its Fap2 protein, thereby inhibiting the cytotoxic functions of NK cells and CD8^+^ T cells (Gur et al., 2015). It is also associated with the enrichment of M2-like tumor-associated macrophages (TAMs), regulatory T cells (Tregs), and myeloid-derived suppressor cells (MDSCs), thus promoting the formation of an immune-evasive microenvironment (Kostic et al., 2013; Jiang et al., 2023). *Porphyromonas gingivalis* may promote T-cell exhaustion and weaken anti-tumor immune responses by enhancing NF-κB inflammatory signaling and upregulating PD-L1 expression (Wang M. et al., 2023; Wang et al., 2024a). In contrast, some studies suggest that LCC has relatively lower immune infiltration, but its local immune status may be more strongly influenced by bacteria producing short-chain fatty acids (SCFAs) and their metabolites. SCFA-producing bacteria such as *Faecalibacterium prausnitzii*, *Roseburia*, and *Bacteroides* can regulate inflammatory responses and T-cell function through the GPR43 pathway, HDAC inhibition, and inflammasome-related pathways, thereby contributing to intestinal immune homeostasis (Fusco et al., 2023; Thulasinathan et al., 2025). Bifidobacterium and *Akkermansia muciniphila* can enhance antigen presentation and T-cell immune responses, thereby improving tumor sensitivity to immunotherapy (Dong et al., 2020; Gao et al., 2023). In addition, gut microbial metabolites also exhibit site-specific differences. Although butyrate-producing bacteria can be detected in the left colon, SCFA levels are generally lower than those in the right colon, because fermentable substrates such as dietary fiber are largely utilized in the proximal colon (Cong et al., 2024). However, current understanding of these region-specific metabolic differences is still largely based on experimental systems and mouse models, and whether these findings can fully reflect metabolic conditions in the human colon remains unclear. Given that microbiota-related mechanisms involve multilayered regulation of inflammation, immunity, barrier function, and metabolism, single-target interventions may not fully address this complex network. Therefore, multi-target and systematic interventions through microbiota modulation may provide a new direction for individualized CC treatment. Moreover, the holistic regulatory characteristics of TCM offer a theoretical entry point for further exploring its microbiota-mediated effects.

## 5 Efficacy and mechanisms of TCM in CC through gut microbiota regulation

### 5.1 Clinical connotations and modern interpretation of TCM syndromes in CC

TCM syndromes are a core component of the TCM system of syndrome differentiation and treatment. They refer to the features of pathogenesis at a specific stage of a disease, summarized based on symptoms, signs, and the overall condition of the patient during disease development and progression. Therefore, syndromes can reflect differences in disease stage, host status, and therapeutic response. Syndrome identification is an important basis for individualized TCM treatment (Bao et al., 2010; Jiang et al., 2012).

TCM syndromes involve theoretical concepts such as “qi and blood,” “yin and yang,” “healthy qi and pathogenic factors,” and “stasis-toxin.” However, these concepts currently lack unified, precise, and quantifiable definitions within modern biomedical frameworks. Therefore, this review does not directly equate these concepts with any specific molecule, immune cell, microbial taxon, metabolite, or signaling pathway. Instead, it discusses their potential links with the gut microbiota, metabolic abnormalities, inflammatory responses, and the immune microenvironment from functional and correlative perspectives.

RCC and LCC differ markedly in anatomical structure, molecular features, immune status, and spatial microbial distribution, suggesting that CC arising from different anatomical sites may have distinct biological bases. TCM syndromes are dynamically evolving and may also be associated with host status, microbial composition, metabolic environment, and immune-inflammatory state (Lu et al., 2012). Therefore, exploring TCM syndromes in CC from the perspective of the gut microbiota may help deepen our understanding of the biological basis of syndrome differences and provide insights for bridging TCM syndrome differentiation with modern tumor microbiome research.

### 5.2 Major TCM syndrome patterns in CC and their clinical features

In TCM literature, CC is classified under categories such as “intestinal polypoid mass” (*Chang Xun*), “abdominal mass” (*Ji Ju*), and “intestinal bleeding” (*Chang Feng*). Among these, the term *Chang Xun* first appeared in *Lingshu · Shui Zhang*, which described a pathological process involving the invasion of cold pathogens into the intestines, impaired qi movement, internal accumulation of pathogenic factors, and the subsequent formation of intestinal masses. This description provides an early theoretical basis for understanding the disease location and pathogenesis of CC in TCM, although ancient physicians did not distinguish between RCC and LCC according to modern anatomical concepts.

It should be noted that although RCC and LCC differ in anatomical location, clinical manifestations, molecular features, and microbial ecology, TCM syndromes are not mechanically classified based solely on these differences. Instead, syndrome differentiation also requires comprehensive consideration of patient constitution, disease stage, symptom presentation, and the dynamic evolution of pathogenesis. Moreover, there is currently insufficient evidence to support a fixed correspondence between any specific TCM syndrome and one particular side of the colon. Therefore, based on the overall syndrome characteristics of CC and with reference to the *2024 Chinese Guidelines for Integrated Diagnosis and Treatment of Malignant Tumors: Colon Cancer Section* (Chinese Anti-Cancer Association and Committee of Colorectal Cancer of Chinese Anti-Cancer Association, 2025), this review discusses six common TCM syndromes: spleen-stomach disharmony syndrome, qi-blood deficiency syndrome, spleen-kidney yang deficiency syndrome, blood stasis and toxin obstruction syndrome, dampness-heat toxin syndrome, and yin deficiency of liver and kidney syndrome. Based on the above classification, Table 4 provides a concise summary of the pathogenesis and clinical characteristics of these six prevalent TCM syndrome patterns in CC, thereby facilitating a more structured understanding of their clinical features.

**TABLE 4:** Pathogenesis and clinical characteristics of prevalent TCM syndromes in CC.

TCM syndrome	Pathogenesis	Clinical characteristics
Spleen-stomach disharmony	Dysfunction of the middle jiao, leading to impaired qi movement and digestive dysfunction	Epigastric fullness, anorexia, nausea, vomiting, abdominal distension, and diarrhea
Qi and blood deficiency	Chronic spleen-stomach deficiency, leading to impaired generation of qi and blood	Pale lips and nails, shortness of breath, fatigue, anorexia, poor appetite, and dull abdominal pain
Spleen-kidney Yang deficiency	Prolonged spleen yang deficiency, gradually involving kidney yang and leading to internal cold and impaired intestinal function	Chronic diarrhea, dull abdominal pain, cold intolerance, cold limbs, and lethargy
Blood stasis and toxin obstruction syndrome	Qi stagnation, blood stasis, accumulation of toxic pathogens, and injury to intestinal collaterals	Abdominal pain, hematochezia, anemia, and a dusky complexion
Damp-heat toxin	Dietary irregularities leading to damp-heat accumulation and toxin retention in the intestinal tract	Abdominal pain and distension, tenesmus, mucous stools, and hematochezia
Yin deficiency of liver and kidney	Prolonged illness consuming yin fluids, leading to intestinal dryness and loss of nourishment	Emaciation, constipation, intestinal obstruction, and palmoplantar heat sensation with dysphoria

### 5.3 Potential associations of TCM syndromes with gut microbiota, metabolites, and immune pathways

Wang and colleagues (Wang et al., 2020) performed 16S rRNA sequencing of fecal samples from CRC patients with different TCM syndromes and preliminarily revealed a potential biological association between gut microbiota alterations and TCM syndrome differentiation. Their findings suggested that different syndromes, such as excess toxin accumulation syndrome and healthy qi deficiency syndrome, exhibited distinct microbial community characteristics. Using linear discriminant analysis effect size (LEfSe) analysis, Gui et al. (2023) further identified representative microbial taxa associated with different TCM syndromes, which may serve as potential biomarkers for syndrome differentiation in CC and may assist in more accurate syndrome classification for individualized TCM treatment. However, it should be noted that current evidence is largely based on associative findings, and the causal relationships between specific syndromes and particular microbial taxa, metabolites, or signaling pathways remain to be further validated.

According to TCM theory, factors such as exogenous pathogenic influences, emotional disorders, improper diet, and imbalance between work and rest may impair healthy qi and contribute to disease occurrence. From a modern medical perspective, the gut microbiota is influenced by multiple factors, including age (Grieneisen et al., 2021), dietary preferences (Da Ros et al., 2021), disease status (Yeoh et al., 2021), and drug exposure (Klünemann et al., 2021). These factors may also participate in CC progression by altering intestinal barrier integrity, microbial composition, immune-inflammatory responses, and the metabolic environment, suggesting potential parallels between TCM pathogenesis theory and modern microbiome-related mechanisms.

Previous studies have shown that under conditions of relatively stable gut microbial ecology and intact host immune function, beneficial bacteria such as *Lactobacillus* and their metabolites are relatively enriched. Multiple *in vivo* studies suggest that these bacteria may help suppress CC growth and metastasis by promoting CD8^+^ T-cell recruitment (Zhang S.-L. et al., 2022), regulating redox balance (Bell et al., 2022), and stimulating dendritic cells to produce IL-12a and activate anti-tumor immune responses (Zhang W. et al., 2023). Although experimental studies suggest potential anti-tumor effects of health-associated microbial metabolites, whether these metabolites can achieve biologically meaningful concentrations *in vivo* remains uncertain. In contrast, when gut microbial homeostasis is disrupted and accompanied by abnormal host immune regulation, *Lactobacillus* abundance decreases while potentially pathogenic bacteria such as *F. nucleatum* become enriched. *In vitro* and *in vivo* studies have shown that these pathogenic bacteria may remodel the intestinal microenvironment and increase the risk of CC invasion and metastasis through modulation of Th17 responses (Zhang Q. et al., 2023), activation of the NF-κB signaling pathway, promotion of glucose metabolism in CC cells (Brennan et al., 2021), and induction of aryl hydrocarbon receptor (AHR) signaling by microbial metabolites such as formate (Ternes et al., 2023). These findings suggest that CC-related TCM syndromes should not be regarded as fixed states, but rather as dynamic processes that may evolve along with changes in host immunity, microbial composition, metabolic environment, and tumor progression stage (Wang et al., 2020).

Based on the studies described above, this review further summarizes common TCM syndromes in CC, including their associated microbial and metabolic characteristics, potential biological mechanisms, and possible links with differences between RCC and LCC, together with the corresponding study populations, experimental models, and sources of evidence, as presented in Table 5. It should be emphasized that the contents of the table are primarily intended to facilitate understanding of syndrome-related pathogenic features from the perspectives of the gut microbiota, associated metabolic alterations, and microbiota-mediated immune-inflammatory and molecular pathways. Interpretations involving differences between RCC and LCC are mainly exploratory analyses based on current evidence and TCM theory, and do not imply a fixed correspondence between any specific TCM syndrome and a particular tumor location.

**TABLE 5:** Gut microbiota signatures, mechanisms, and anatomical heterogeneity associated with TCM syndromes in CC.

TCM Syndrome	Microbiota and Metabolic Features	Potential Biological Mechanisms	TCM Pathogenesis Interpretation	Potential Association with RCC/LCC Differences	Study Subjects/Models	References (PMID)
Spleen-stomach disharmony syndrome	*Selenomonadaceae* ↑; pro-inflammatory microbial shift	Intestinal inflammation; epithelial oxygenation ↑; anaerobic niche disruption; facultative anaerobe expansion	1) Dysfunction of ascending and descending activities in the middle jiao 2) Impaired transformation and transportation of food and fluids 3) Disturbed distribution of nutrients and impaired waste excretion 4) Possible association with persistent intestinal inflammation and microenvironmental imbalance	The right colon is mainly involved in water and electrolyte absorption. From a TCM functional perspective, symptoms commonly observed in RCC, such as abdominal pain, bloating, or loose stools, may partly be understood in this context; however, this does not indicate a fixed correspondence between spleen–stomach disharmony syndrome and RCC	Preoperative fecal samples from CRC patients	Gui et al. (2023)
	Facultative anaerobes ↑; certain obligate anaerobes ↓	Barrier injury; altered epithelial oxygen metabolism; weakened obligate anaerobe colonization; dysbiosis–inflammation cycle			Review studies	Wang et al. (2011), Litvak et al. (2017), Shah and Itzkowitz (2022), Zhang et al. (2024)
Qi and blood deficiency syndrome	*A. muciniphila* participates in anti-tumor immune regulation	*Inhibition of colorectal tumorigenesis; M1-like macrophage infiltration ↑*	1) Deficiency of healthy qi leads to reduced resistance against pathogenic factors 2) The spleen and stomach are regarded as the source of qi and blood generation 3) Prolonged spleen deficiency may result in insufficiency of qi and blood 4) Malnourishment of the intestinal tract and impaired defense and repair capacity may occur	The right colon is anatomically located on the right side and, in TCM theory, is considered more prone to concealed onset. Clinical manifestations such as chronic blood loss, anemia, fatigue, and emaciation are relatively common in RCC and may partly be interpreted from the perspective of qi–blood deficiency; however, this does not indicate a fixed correspondence between RCC and qi–blood deficiency syndrome	Clinical CRC cohorts and GMrepo database; HCT116 and CT26 xenograft mouse models; *in vivo* and *in vitro* macrophage experiments	Fan et al. (2021), Yang et al. (2021), Lin et al. (2023)
	Probiotic intervention is associated with host immune status	Probiotics plus IL-2 may enhance antitumor immune responses				
	Reduced abundance of *A. muciniphila* may be associated with impaired barrier function and weakened immune defense	TLR2/NLRP3 and NF-κB-related inflammatory regulation; intestinal barrier impairment			CRC patient-derived tumor tissues; B16F10 melanoma-bearing mice; CT26 colon cancer-bearing mice	Shi et al. (2020)
Spleen-kidney Yang deficiency syndrome	SCFA-related metabolic function ↓ in advanced deficiency syndromes	Energy metabolism abnormality; intestinal functional decline	1) Prolonged spleen yang deficiency and chronic disease progression may further impair kidney yang 2) Deficiency of kidney yang may weaken warming, promoting, and transforming functions 3) Clinical manifestations may include chronic diarrhea, concealed abdominal pain, cold intolerance, and fatigue	The right colon is characterized by active water/electrolyte absorption and microbial fermentation. Reduced SCFAs, insufficient epithelial energy supply, and weakened intestinal barrier function may partly correspond to the deficiency-cold features observed in some RCC patients with prolonged disease courses; however, this should not be interpreted as a fixed correspondence with RCC	Preoperative fecal samples from CRC patients	Gui et al. (2023)
	Opportunistic pathogens such as *Campylobacter ureolyticus* ↑	May be associated with CpG island methylator phenotype (CIMP) in colorectal cancer			Correlation studies	Park et al. (2024)
	PKS island-carrying bacteria; colibactin-producing strains	Genotoxin production; possible association with CIMP			CRC tumor tissues and adjacent normal tissues; TCGA database	Kaur et al. (2023)
Blood stasis and toxin obstruction syndrome	Toxin-associated bacteria and metabolites ↑: *Bacteroides fragilis*, *Escherichia coli*-related endotoxins, secondary bile acids, N-nitroso compounds, TMAO, ethanol, and hydrogen sulfide	DNA damage; epithelial barrier disruption; tumor-promoting signaling activation; chronic inflammation; local pro-tumor microenvironment formation; *B. fragilis* toxin-mediated E-cadherin cleavage and β-catenin/NF-κB activation	1) Prolonged disease progression may lead to impaired qi movement 2) Poor blood circulation and persistent retention of pathogenic toxins may occur within the intestinal collaterals 3) These changes may contribute to sustained local lesion progression 4) This may share pathological similarities with toxin accumulation, intestinal collateral injury, and progressive tumor development	The left colon is more involved in fecal storage, propulsion, and excretion, and the intestinal contents are relatively more formed, making constipation, difficult defecation, obstruction, and hematochezia more likely to occur. From the perspective of the TCM functional concept of “left ascending and right descending,” impaired qi movement and poor blood circulation may contribute to abnormal intestinal transit and the accumulation of stasis and pathogenic toxins. Although stasis–toxicity obstruction syndrome may be observed in both RCC and LCC, LCC may more readily reflect pathogenic features associated with qi stagnation, impaired bowel passage, and obstruction of the intestinal collaterals by stasis and toxins	Review studies	Mohseni et al. (2020)
	PKS island-carrying *E. coli*, colibactin-producing strains	DNA damage; epithelial injury; tumor-promoting signaling activation; chronic inflammation; pro-tumor microenvironment formation			CC xenograft mouse models, azoxymethane/dextran sulfate sodium (AOM/DSS)-induced mouse models, and intestinal epithelial cell experiments	Dalmasso et al. (2014)
	Colibactin-associated genotoxicity	DNA alkylation; double-strand breaks; SENP1-related pathway activation; tumor proliferation			Cancer genomics cohorts	Pleguezuelos-Manzano et al. (2020)
Damp-heat toxin syndrome	Complex interactions exist among gut microbiota, chronic inflammation, and tumor development	Dysbiosis-associated inflammation and tumor promotion	1) Improper diet and preference for greasy or rich foods may impair spleen–stomach transformation and transportation 2) Qi stagnation, dampness accumulation, and prolonged heat-toxin retention may occur in the intestine 3) These pathological changes may manifest as hematochezia and mucus-containing stools	The left colon is more involved in fecal storage, propulsion, and excretion. From the perspective of TCM image-based thinking, in which “the left pertains to yang and yang governs qi transformation,” microbiota dysbiosis, barrier injury, and persistent inflammatory activation associated with high-fat/high-sugar diets may provide a modern biological reference for damp–heat toxin-related pathogenic features in LCC	Review studies	Brennan and Garrett (2016)
	High-fat/high-sugar diets; intestinal mucosal *E. coli* colonization ↑	Mucus layer thinning; intestinal permeability ↑; mucosal inflammation ↑; intestinal homeostasis disruption			High-fat/high-sugar diet-induced mouse models	Martinez-Medina et al. (2014)
	High-fat diets: *Alistipes* spp. ↑; *Parabacteroides distasonis* and *Parabacteroides* sp. CT06 ↓; LPA and LPC ↑	CRC cell growth ↑; organoid proliferation ↑; cell-cycle progression; tight junction protein expression ↓			AOM-induced CRC mouse models; *Apc* ^min/+^ mouse models; germ-free mouse fecal microbiota transplantation models; CRC cell lines and patient-derived CRC organoid experiments	Yang W. et al. (2022)
Yin deficiency of liver and kidney syndrome	Gut microbiota dysbiosis; metabolic abnormalities; gut–liver axis involvement	Bidirectional gut–liver communication; altered hepatic lipid and bile acid metabolism; inflammation; impaired barrier homeostasis	1) Emotional dysregulation and impaired liver qi dispersion may occur 2) Prolonged qi stagnation may transform into heat 3) Heat may gradually consume liver–kidney yin 4) Intestinal dryness and internal deficiency heat may subsequently develop	Intestinal contents in the left colon become more formed because of water reabsorption. Impaired intestinal nourishment may therefore increase the tendency toward constipation, difficult defecation, and obstruction. Persistent deficiency heat may also contribute to sustained local inflammation and oxidative stress. Thus, liver–kidney yin deficiency syndrome may more readily reflect certain pathogenic features observed in some LCC patients; however, this does not indicate a fixed correspondence with LCC	Review studies	Brennan and Garrett (2016)
	Chronic stress-related gut microbial alteration; chronic low-grade inflammation	Systemic immune alteration; gut microbiota remodeling; chronic inflammatory activation			Chronic and recurrent stress mouse models	Westfall et al. (2021)
	Depression/anxiety-related brain–gut axis immune alterations	Peripheral IL-6 ↑; MHC II^+^CD11c^+^ dendritic cells and Ly6C^hi^ monocytes activation			Repeated social defeat stress mouse models; chronic social defeat stress (CSDS) mouse models combined with bone marrow transplantation experiments	Hodes et al. (2014), Ambrée et al. (2018)
	Weakly agonistic LPS involved in intestinal immune homeostasis	Competitive TLR4 signaling regulation; excessive pro-inflammatory factor release ↓; immune homeostasis restoration			T cell-mediated chronic colitis mouse models; intestinal lamina propria CD11c^+^ cell-associated immune mechanism studies	Steimle et al. (2019)

### 5.4 Research progress and mechanisms of TCM-mediated gut microbiota regulation in CC

**FIGURE 1:**
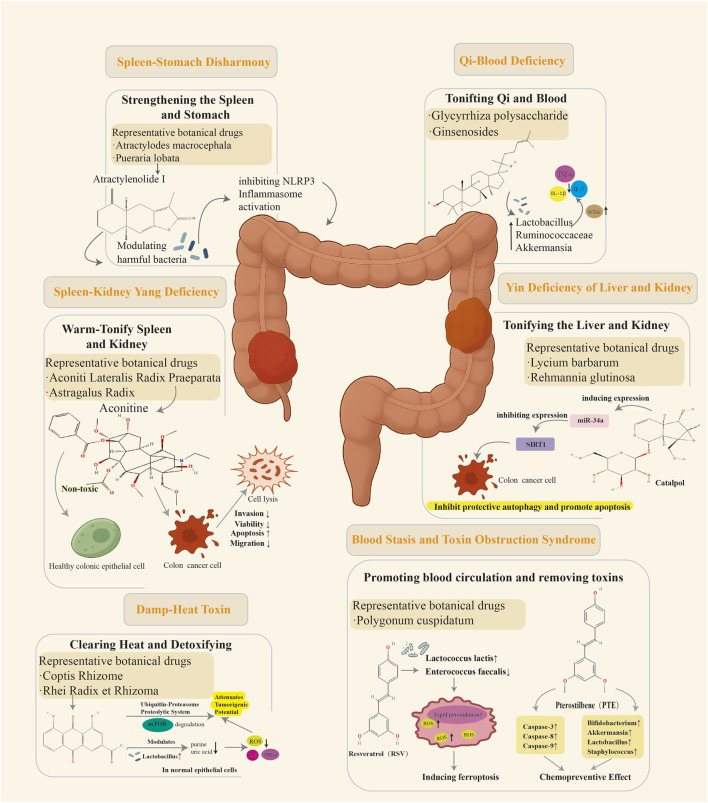
Potential microbiota-associated mechanisms of TCM syndrome-based interventions in colon cancer. This schematic summarizes representative Traditional Chinese Medicine (TCM) syndromes, related botanical drugs, selected active compounds, gut microbiota alterations, and proposed mechanisms involved in colon cancer (CC). For spleen–stomach disharmony syndrome, atractylenolide I derived from Atractylodes macrocephala is shown in relation to inhibition of NLRP3 inflammasome activation and modulation of pathogenic bacteria. For qi–blood deficiency syndrome, ginsenosides and glycyrrhiza polysaccharide are associated with enrichment of beneficial bacteria, increased short-chain fatty acids (SCFAs), and potential immunomodulatory effects. For spleen–kidney yang deficiency syndrome, processed aconite- and astragalus-related compounds are presented in relation to potential effects on tumor cell apoptosis, invasion, migration, and intestinal microbial regulation. For dampness–heat toxin accumulation syndrome, berberine from Coptis chinensis and rhein from rhubarb are shown in relation to pathogenic bacteria reduction, mTOR-related regulation, oxidative stress, and inflammatory responses. For yin deficiency of liver and kidney syndrome, Lycium barbarum polysaccharide is associated with microbiota and SCFA regulation, whereas catalpol from Rehmannia glutinosa is shown in relation to miR-34a/SIRT1-mediated inhibition of protective autophagy and promotion of apoptosis. For blood stasis and toxin obstruction syndrome, resveratrol and pterostilbene from Polygonum cuspidatum are shown in relation to modulation of Lactococcus lactis, *Enterococcus faecalis*, ferroptosis, apoptosis-related markers, and inflammatory regulation. The mechanisms summarized in this figure are mainly derived from preclinical and mechanistic studies and should not be interpreted as fixed correspondences between specific TCM syndromes and tumor sidedness.

TCM-based interventions targeting gut microbiota regulation may provide potential complementary strategies for the prevention and treatment of CC. The potential relationships among representative TCM syndromes, commonly used botanical drugs, microbiota-related alterations, and proposed anti-tumor mechanisms are summarized in Figure 1. After oral administration, the pharmacological effects of TCM are influenced by the gut microbiota. Commensal microorganisms in the intestine encode various enzymes capable of chemically modifying orally administered drugs and their metabolites, thereby affecting drug activity, toxicity, stability, bioavailability, and excretion rate (Pant et al., 2023). Therefore, TCM–microbiota interactions constitute an important aspect of understanding TCM-based interventions in CC. Although RCC and LCC differ in multiple aspects, TCM treatment should still be centered on syndrome differentiation and individualized therapy based on patient constitution, disease stage, syndrome characteristics, and accompanying pathogenic factors, rather than establishing fixed correspondences among botanical prescriptions, TCM syndromes, and the anatomical location of CC.

For spleen-stomach disharmony syndrome, botanical drugs that strengthen the spleen and harmonize the stomach, such as *Atractylodes macrocephala* and *Pueraria lobata*, may be applied according to syndrome differentiation. *Atractylodes macrocephala* is traditionally used to tonify qi and strengthen the spleen. Its active monomer, atractylenolide I, can inhibit NLRP3 inflammasome activation in tumors by suppressing Drp1-mediated mitochondrial fission, thereby improving the inflammatory microenvironment and delaying CC progression (Qin et al., 2021). Animal experiments further demonstrated that in a male BALB/c mouse cancer cachexia model induced by inoculation with murine C26 colon adenocarcinoma cells, treatment with atractylenolide I significantly alleviated progressive body weight loss and characteristic tissue atrophy, including atrophy of muscle, adipose tissue, spleen, and thymus (Zhang et al., 2020). In addition, atractylenolide I reduced intestinal microenvironmental pH, enhanced adhesion of *Bifidobacterium* to intestinal epithelial cells, supported beneficial gut microbiota, inhibited the proliferation of harmful bacteria such as *Escherichia coli* and *Enterococcus faecalis*, and helped maintain intestinal barrier integrity (Shu et al., 2017). *Pueraria lobata* is traditionally used to raise yang and relieve diarrhea. Its major active metabolite, puerarin, can reduce the abundance of *Alistipes*, *Prevotella copri (P. copri)*, and *Veillonella*, while increasing the level of *Desulfovibrionaceae* (Zou et al., 2023). Puerarin also exerts anti-inflammatory and antioxidant effects by decreasing IL-1β, IL-6, and TNF-α levels, downregulating NF-κB signaling, activating the NF-E2-related factor 2 (Nrf2) pathway, and enhancing tight junction protein expression to improve intestinal epithelial barrier dysfunction (Jeon et al., 2020).

For qi-blood deficiency syndrome, botanical drugs that tonify qi and nourish blood, such as ginseng and licorice, are commonly applied. Ginseng strongly tonifies primordial qi and nourishes body fluids and blood. Ginsenosides can increase gut microbiota diversity, enrich beneficial bacteria such as *A. muciniphila*, *Lactobacillus*, and *Akkermansia*, and significantly reduce harmful bacteria including *Odoribacter*, *Clostridia_UCG-014*, and *Bacteroides* (Cheng et al., 2022; Bai et al., 2025). Although these recent studies provide relatively comprehensive mechanistic evidence, the current findings remain largely preclinical. Their clinical safety, effective dosage, and reproducibility in human populations require further validation. These changes may suppress inflammatory cytokines, attenuate pro-inflammatory responses, increase metabolites such as linoleic acid and SCFAs, protect intestinal mucosa, and exert anti-tumor effects (Thamphiwatana et al., 2014; Lyu et al., 2023). Licorice is traditionally used to tonify the spleen and replenish qi. Its active metabolite, glycyrrhiza polysaccharide (GCP), can increase Firmicutes-associated taxa, including *Lachnospiraceae_UCG_001*, *Ruminococcaceae_UCG_010*, and *Ruminococcaceae_UCG_014*, thereby accelerating fiber degradation and promoting butyrate production (Zhang J. et al., 2018). *In vitro*, GCP upregulated IL-7 expression and secretion in intestinal epithelial cells and promoted T-lymphocyte proliferation, suggesting a potential immunomodulatory role related to antitumor immunity (Ayeka et al., 2016).

For spleen-kidney yang deficiency syndrome, botanical drugs that tonify the spleen and kidney and warm yang are commonly used, including processed aconite and astragalus. Properly processed and rationally administered aconite can warm yang and dispel cold. Its major active metabolite, aconitine, does not exhibit significant cytotoxicity toward normal colonic mucosal cells but can induce tumor cell inactivation and apoptosis while inhibiting CC cell migration and invasion (Li et al., 2024a). Therefore, aconite-related metabolites may possess potential anti-tumor value, although their safety, dosage range, and clinical applicability still require rigorous evaluation. Astragalus is traditionally used to tonify qi and raise yang. Astragalus polysaccharides can increase microbial diversity, elevate the abundance of beneficial bacteria such as *Bifidobacterium* and *Lactobacillus*, and reduce harmful bacteria including *Enterococcus faecalis*, *Bacteroides*, and *Clostridium butyricum* (Liu et al., 2021). SCFAs produced by *Bifidobacterium* can activate immune cells, regulate second messenger systems, control intestinal mucosal inflammation, inhibit tumor growth and differentiation, and promote tumor apoptosis, thereby suppressing CC progression (Dong et al., 2023).

For dampness-heat toxin accumulation syndrome, botanical drugs that clear heat, eliminate dampness, and detoxify are commonly prescribed, such as *Coptis chinensis* and rhubarb. *Coptis chinensis* possesses strong heat-clearing and detoxifying effects. Berberine, its major active metabolite, can downregulate the Hedgehog signaling pathway in CC cells while reducing pathogenic bacteria such as *F. nucleatum*, thereby promoting degradation of KIF26B mRNA and inhibiting CC cell proliferation, metastasis, and colony formation (Chen F. et al., 2022; Sun et al., 2022). Rhubarb is traditionally used to purge accumulation and clear heat toxins. Rhein, a natural anthraquinone metabolite extracted from rhubarb, promotes mTOR degradation through the ubiquitin–proteasome pathway and can directly bind to mTOR to inhibit mTOR signaling. Rhein can also increase the abundance of *Lactobacillus* (Wu et al., 2020), thereby reducing intestinal purine and uric acid accumulation, oxidative stress, and inflammatory responses associated with these metabolites, ultimately lowering CC risk. In xenograft mouse models, rhein exhibited low toxicity and potent anti-tumor activity, suggesting broad therapeutic potential (Zhang et al., 2021).

For blood stasis and toxin obstruction syndrome, botanical drugs that activate blood circulation and detoxify, such as *Polygonum cuspidatum* and *Panax notoginseng*, are often applied. *Polygonum cuspidatum* promotes blood circulation and clears heat toxins and is an important source of resveratrol (RSV). Zhang et al. demonstrated in an HT29 xenograft female mouse model that RSV promoted the growth of *Lactococcus lactis* while inhibiting *Enterococcus faecalis*, thereby maintaining intestinal homeostasis. RSV also increased reactive oxygen species and lipid peroxidation in CC cells, ultimately inducing ferroptosis (Wang Z. et al., 2022; Zhang Z. et al., 2022). However, these findings should be interpreted with caution, as the observed antitumor effects were achieved using a materially optimized nano-delivery system and therefore cannot be directly extrapolated to the clinical effects of RSV derived from botanical drugs or ordinary Polygonum cuspidatum preparations. In addition, pterostilbene, a methoxylated derivative of RSV, can effectively reduce oxidative stress and inflammatory responses, increase apoptosis-related parameters including caspase-3, caspase-8, and caspase-9, decrease the abundance of *Staphylococcus*, and increase beneficial bacteria such as *Bifidobacterium*, *Akkermansia*, and *Lactobacillus*, thereby exerting chemoprotective effects against CC (Guo C. et al., 2025).

For yin deficiency of liver and kidney syndrome, botanical drugs that nourish the liver and kidney and clear deficiency heat, such as wolfberry and *Rehmannia glutinosa*, are commonly used. Wolfberry nourishes liver-kidney yin and essence-blood. *Lycium barbarum* polysaccharide (LBP), its major active metabolite, can downregulate PMI and ABCG2 expression, inhibit tumor cell proliferation and migration, and regulate the PI3K/AKT pathway, thereby potentially reversing chemoresistance in CC cells (Ma et al., 2024). LBP can also enrich beneficial bacteria including *Ruminococcaceae*, *Lactobacillus*, *Butyricicoccus*, and *Akkermansia*, increase SCFA production, and alleviate intestinal inflammation (Li et al., 2023). *Rehmannia glutinosa* clears heat, nourishes yin, and generates body fluids. Its active monomer catalpol exhibits anti-inflammatory, antioxidant, and anti-proliferative properties (Liu J. et al., 2023). Catalpol can inhibit protective autophagy and promote apoptosis in CC cells by inducing miR-34a expression and directly suppressing the downstream target protein *SIRT1*, thereby attenuating malignant behavior (Qiao et al., 2020). Catalpol may also reverse the Firmicutes/Bacteroidetes (F/B) ratio, increase butyrate-producing bacteria, and reduce the predominance of pro-inflammatory microbiota, thereby contributing to CC prevention and treatment (Zhang W. et al., 2023).

In addition to single botanical drugs and their active metabolites, classical TCM formulas also represent important approaches for syndrome-based CC intervention. TCM treatment of CC should be based on syndrome differentiation and tailored according to syndrome characteristics and pathogenic evolution, including methods such as strengthening the spleen and harmonizing the stomach, tonifying qi and blood, warming the spleen and kidney, clearing heat and detoxifying, activating blood circulation, and nourishing the liver and kidney. Representative formulas discussed in this review include Sijunzi Decoction, Bazhen Decoction, Sishen Decoction, Taohong Siwu Decoction, Huanglian Jiedu Decoction, and Liuwei Dihuang Pill. Their compositions, therapeutic principles, commonly applicable pathogenic features, and current microbiota-related evidence are summarized in Table 6. It should be emphasized that the contents listed in Table 6 are intended only to summarize existing evidence and syndrome differentiation strategies and should not be interpreted as fixed prescription patterns.

**TABLE 6:** Representative TCM formulas for colon cancer intervention and modern evidence.

Botanical Formula	Composition	Main Effects	Modern Research	Study Subjects/Models	References (PMID)
Sijunzi decoction (SJZD)	*Panax ginseng*, *Atractylodes macrocephala*, *Poria cocos*, and *Glycyrrhiza uralensis*	Qi-tonifying and spleen-supporting effects	SJZD increased the sensitivity of CC cells to NK cell-mediated cytotoxicity by regulating P53 expression and upregulating DR4 and DR5	*In vitro* experiments involving CC cells and NK cells	Wang et al. (2024b)
			SJZD significantly enriched beneficial bacteria, such as *Lactobacillus johnsonii* and *Lactobacillus taiwanensis,* providing evidence for its potential role in improving gut microbial balance and intestinal barrier function	Spleen deficiency-related animal models; multi-omics analysis	Dong et al. (2024)
Bazhen decoction (BZD)	*Panax ginseng*, *Atractylodes macrocephala*, *Poria cocos*, *Glycyrrhiza uralensis*, *Rehmannia glutinosa*, *Angelica sinensis*, *Paeonia lactiflora*, and *Ligusticum chuanxiong*	Qi and blood replenishment	BZD may improve the tumor microenvironment and inhibit CC progression by regulating the PI3K-AKT, P53, and VEGF pathways, as well as the expression of PI3K, AKT, MYC, and EGFR. It may also promote T-cell infiltration and activation, reduce PD-1 expression, restore T-cell cytotoxicity, and enhance antitumor immunity	Network pharmacology and molecular docking; CRC cell experiments; MC38 tumor-bearing mouse model	Ersahin et al. (2015), Zhao et al. (2019), Lu et al. (2023)
Sishen decoction (SSD)	*Psoralea corylifolia*, *Myristica fragrans*, *Evodia rutaecarpa*, and *Schisandra chinensis*	Warming the kidney and spleen, astringing the intestine, and relieving diarrhea a	SSD promoted autophagy, inhibited endoplasmic reticulum stress, increased MUC2 and sIgA levels, and reduced IL-6 expression, thereby improving intestinal inflammation and barrier injury in kidney-yang deficiency diarrhea mice. It also modulated the gut microbiota by enriching beneficial bacteria such as *Lactobacillus* and *Clostridium*, reducing harmful bacteria including *Porphyromonas* and *Parabacteroides*, and promoting SCFA production, including propionate and butyrate	Kidney-yang deficiency diarrhea mouse model; IBS-D mouse model	Li et al. (2024b), Zhao et al. (2024), Guo M. et al. (2025)
Taohong Siwu decoction (THSWD)	*Prunus persica*, *Carthamus tinctorius*, *Angelica sinensis*, *Ligusticum chuanxiong*, *Paeonia lactiflora*, and *Rehmannia glutinosa*	Promoting blood circulation, removing blood stasis, and nourishing blood	Significant phenotypic differences in glioma cells were observed between germ-free mouse serum and normal mouse serum. Serum from THSWD-fed mice inhibited tumor cell proliferation, induced autophagy, and downregulated CDC6 pathway activity, suggesting that the antitumor effects of blood-activating formulas may be regulated by microbial metabolism	Serum from THSWD-fed mice; germ-free/normal mouse serum comparison; glioma cell experiments	Feng et al. (2023)
Huanglian Jiedu decoction (HLJDD)	*Coptis chinensis*, *Scutellaria baicalensis*, *Phellodendron chinense*, and *Gardenia jasminoides*	Clearing heat, detoxifying, purging fire, and drying dampness	HLJDD alleviated chemotherapy-induced diarrhea by protecting the intestinal mucosa, promoting epithelial repair, and maintaining CD44^+^ intestinal stem cells and Wnt/β-catenin signaling	5-FU- and CPT-11-induced chemotherapy-associated diarrhea mouse models	Su et al. (2016), Zhang X. et al. (2018)
			HLJDD enhanced the antitumor effects of FOLFOX/FOLFIRI in orthotopic colorectal cancer mouse models	Orthotopic colorectal cancer mouse models treated with FOLFOX/FOLFIRI	Chan et al. (2020)
Gegen qinlian decoction (GGQLD)	*Pueraria lobata*, *Scutellaria baicalensis*, *Coptis chinensis*, and *honey-fried Glycyrrhiza uralensis*	Releasing the exterior, clearing internal heat, and relieving dysentery	GGQLD regulated cell cycle, focal adhesion, and NOD-like receptor signaling pathways associated with oncogene and tumor suppressor gene dysregulation. It also increased CD8^+^ T-cell infiltration, elevated IL-2 and IFN-γ levels, reduced PD-1-related immunosuppression, and enhanced anti-PD-1 therapy by remodeling the gut microbiota and tumor immune microenvironment	GGQLD enriched beneficial bacteria such as *coprococcu*s, *Bifidobacterium*, *Blautia,* and *Akkermansia*, improved lipid metabolism, and reduced carcinogenesis risk	Lv et al. (2019)
			Microsatellite-stable CRC mouse model; anti-PD-1 combination therapy; gut microbiota and tumor immune microenvironment analysis	Type 2 diabetic rat model; gut microbiota and metabolite analysis	Xu et al. (2024)
Liuwei Dihuang Pill (LWDHW)	*Rehmannia glutinosa*, *Cornus officinalis*, *Dioscorea opposita*, *Alisma orientale*, *Moutan cortex*, and *Poria cocos*	Nourishing liver and kidney	Long-term intervention with spleen- and kidney-tonifying botanical medicines may reduce the risk of postoperative recurrence and metastasis in patients with stage II–III CRC, and LWDHW was among the most frequently prescribed formulas	Cohort study of patients with stage II–III CRC after radical surgery	Tang et al. (2022)
			LWDHW increased the abundance of intestinal bacteria such as *Lactobacillus*, *Allobaculum*, and *Ruminococcus*_2, elevated SCFA levels, including acetate, propionate, and butyrate, and may improve intestinal metabolism through the SCFA–GPR43/41–GLP-1 pathway	Goto-Kakizaki type 2 diabetic rat model; 16S rDNA sequencing and SCFA analysis	Yi et al. (2022)
Yiyi Fuzi Baijiang powder (YYFZBJS)	*Coix lacryma-jobi*, *processed Aconitum carmichaelii*, and *Patrinia scabiosifolia*	Warming yang, dispersing masses, eliminating dampness and toxins, and draining pus	YYFZBJS inhibited the proliferation, migration, and invasion of CC cells, promoted apoptosis, and reduced xenograft tumor size and weight in nude mice. The underlying mechanisms may involve inhibition of the TLR4/NF-κB pathway, downregulation of TNF-α, IL-1β, and IL-6, reduction of SMOX expression, and alleviation of ROS generation and inflammation-related DNA damage	Network pharmacology; CRC cell experiments; nude mouse xenograft models	Xiang et al. (2022)
			YYFZBJS may regulate the CRC immune-inflammatory microenvironment through the PI3K-Akt, TNF, and IL-17 signaling pathways	Network pharmacology and bioinformatics analysis	Liu Y. et al. (2023)
Pien Tze Huang (PZH)	*Panax notoginseng*, *musk*, *bezoar*, and *snake gall*	Clearing heat and toxins, promoting blood circulation, reducing swelling, and relieving pain	In AOM/DSS-induced CRC and *Apc*min/+ spontaneous intestinal tumor models, PZH dose-dependently reduced tumor incidence, number, and volume, and decreased Ki-67-positive cells; PZH reshaped gut microbiota by enriching beneficial bacteria such as *Pseudobutyrivibrio xylanivorans* and *Eubacterium limosum* while reducing potential pathogens including *Aeromonas veronii*, *Campylobacter jejuni*, *Collinsella aerofaciens*, and *Peptoniphilus harei*; it also regulated taurine metabolism, bile acid metabolism, and unsaturated fatty acid metabolism, improved intestinal barrier function, reduced serum FITC-dextran and LPS levels, upregulated E-cadherin, Occludin, and ZO-1, and inhibited PI3K-Akt, IL-17, TNF, and chemokine-related inflammatory pathways	AOM/DSS-induced CRC mouse models; *Apc* ^min/+^ spontaneous intestinal tumor models; germ-free/antibiotic-treated models; CRC cell and organoid experiments	Kanauchi et al. (2006), Boddu et al. (2015), Wong et al. (2017), Cobo (2018), He et al. (2019), León et al. (2020), Li et al. (2021), Zuo et al. (2021), Gou et al. (2023)
Xiaochaihu decoction (XCHT)	*Bupleurum chinense*, *Scutellaria baicalensis*, Pinellia ternata, *Panax ginseng*, *Glycyrrhiza uralensis*, *ginger*, and *jujube*	Harmonizing Shaoyang and strengthening healthy qi while eliminating pathogenic factors	XCHT may attenuate toxicity and enhance therapeutic efficacy during CPT-11 treatment by alleviating intestinal toxicity through upregulation of ZO-1 and Occludin, restoration of intestinal barrier function, inhibition of the NLRP3 inflammasome pathway, and reduction of inflammatory responses. Meanwhile, XCHT synergized with CPT-11 and SN-38 to promote tumor cell apoptosis by regulating the Bax/Bcl-2 ratio	CPT-11-related intestinal toxicity models; tumor cell apoptosis experiments	Zhang et al. (2026)

Although TCM-based modulation of the gut microbiota demonstrates significant advantages, several problems and challenges remain in clinical application. For example, the potential mechanisms underlying gut microbiota–host interactions have not yet been fully elucidated. The composition of TCM is highly complex, and uncertainties remain regarding its application in CC treatment. A single botanical drug may contain multiple bioactive metabolites, and the specific targets as well as synergistic or antagonistic interactions among these metabolites remain incompletely understood. In addition, considerable individual variability exists, and clinical prescriptions are often tailored according to patient constitution. Furthermore, some patients may be sensitive to specific botanical metabolites, potentially inducing allergic reactions and affecting therapeutic efficacy.

## 6 Discussion and future perspectives

This review systematically summarizes the significant heterogeneity between RCC and LCC in terms of anatomical and physiological characteristics, molecular genetic features, immune microenvironment, and spatial distribution of the gut microbiota, and further discusses the potential role of the gut microbiota in tumor development, immune microenvironment remodeling, and metabolic pathway regulation. Current evidence suggests that spatial heterogeneity of the gut microbiota may represent one of the important factors linking the biological differences, local microenvironmental alterations, and therapeutic response disparities between RCC and LCC. From the perspective of Traditional TCM, the gut microbiota should not be used to mechanically classify TCM syndromes in RCC and LCC. Instead, it may serve as an important modern biological reference for understanding the pathogenesis, symptom characteristics, and individualized TCM interventions in CC arising from different anatomical sites.

In recent years, accumulating evidence has shown that the anti-tumor effects of TCM are closely associated with the gut microbiota. Active metabolites of Chinese botanical medicine can regulate microbial composition and metabolic function. For example, Pien Tze Huang has been shown to increase the abundance of the probiotic bacterium *Pseudobutyrivibrio xylanivorans* and downregulate inflammation-related pathways (Lin et al., 2020); astragalus polysaccharides can promote SCFA production and enhance anti-tumor immunity (Feng et al., 2019); and berberine can delay tumor progression by inhibiting pathogenic bacteria and regulating intestinal barrier function (Shao et al., 2021). Conversely, the gut microbiota can also participate in the metabolic transformation of botanical metabolites through various enzymatic reactions. For instance, rhein may be converted by the gut microbiota into metabolites with higher biological activity (Swanson, 2015). Such bidirectional interactions may collectively influence drug absorption, distribution, efficacy, and safety, and may contribute to comprehensive CC intervention through multiple pathways, including immune regulation, inflammation alleviation, and barrier repair (Walker et al., 2005; Li et al., 2018).

However, this field still faces substantial challenges. First, gut microbiota-mediated drug metabolism may exert a “double-edged sword” effect, as it can either enhance drug activity or lead to loss of efficacy and unpredictable toxic effects (Swanson, 2015). Second, considerable interindividual differences exist in constitution, dietary habits, medication history, and intestinal physiological conditions, including pH, enzymatic activity, motility, and baseline microbial composition. These factors contribute to substantial individual variability in the interactions among Chinese botanical medicine, the gut microbiota, and the host, thereby increasing the difficulty of efficacy evaluation and treatment standardization (Walker et al., 2005; Li et al., 2018). It should also be noted that part of the supporting evidence is derived from *in vitro* fermentation systems, which are still unable to fully recapitulate the complex conditions of the actual colonic lumen. In addition, current studies are still largely limited to animal experiments, *in vitro* mechanistic studies, or cross-sectional microbiota analyses. Well-designed prospective clinical studies remain lacking, and the causal relationship between microbial heterogeneity and the therapeutic effects of TCM interventions has not yet been fully elucidated.

Emerging multi-omics technologies provide important tools for addressing these issues. Metabolomics enables dynamic tracking of interactions between gut microbiota and botanical -derived metabolites. By analyzing the generation, transformation, and distribution of metabolites, researchers may further elucidate the production, conversion, and functional changes of microbiota-related metabolites during TCM intervention and identify key metabolic molecules and potential therapeutic biomarkers associated with anti-tumor effects (Li et al., 2017; Li et al., 2022).

Spatial omics technologies offer novel approaches for understanding the site-specific characteristics and microenvironmental dependence of the gut microbiota. These technologies enable *in situ* analysis of microbial distribution patterns, metabolic activity, and interactions with local immune and tumor cells across different colonic regions (Kneis et al., 2023). This may help further clarify differences between RCC and LCC in microbial distribution, local immune microenvironment, and metabolic characteristics, and provide more precise biological evidence for individualized TCM interventions. Nevertheless, it should be emphasized that site-specific investigations do not imply a fixed correspondence between TCM syndromes or botanical prescriptions and RCC/LCC. Instead, they should contribute to a dynamic understanding of disease mechanisms and treatment-response differences among patients.

Furthermore, the rapid development of single-cell multi-omics technologies enables researchers to analyze the genomic, transcriptomic, and metagenomic characteristics of different cell populations within tumor tissues at single-cell resolution, including tumor cells, immune cells, stromal cells, and associated microbiota (Yachida et al., 2019; Yang J. et al., 2022). Such high-resolution analyses not only help reveal the marked heterogeneity of the CC microenvironment but also facilitate observation of molecular and functional alterations in different cell populations during TCM-mediated modulation of the gut microbiota, thereby providing evidence for further understanding the complex relationships among TCM, the gut microbiota, and the tumor microenvironment.

Future studies should integrate standardized TCM syndrome differentiation, sampling from different colonic sites, fecal and mucosal microbiota sequencing, metabolomics, spatial omics, single-cell multi-omics, and *in vitro* and *in vivo* functional validation to systematically clarify the relationships among TCM intervention-induced microbial alterations, host immune status, and the tumor microenvironment. In addition, prospective clinical studies are needed to establish quantifiable and reproducible efficacy evaluation systems and to elucidate the dynamic relationships among TCM syndromes, microbial characteristics, and therapeutic responses. Overall, spatial heterogeneity of the gut microbiota may provide an important modern biological reference for understanding the pathogenesis of CC and guiding individualized TCM interventions, although its clinical translation still requires higher-quality evidence.

## Glossary

CC: Colon cancer
RCC: right-sided colon cancer
LCC: left-sided colon cancer
TCM: traditional Chinese medicine
CRC: colorectal cancer
CMS: consensus molecular subtypes
MSI-H: microsatellite instability-high
dMMR: deficient mismatch repair
CIMP: CpG island methylator phenotype
CIN: chromosomal instability
ICIs: immune checkpoint inhibitors
DCA: deoxycholic acid
TIGIT: T cell immunoreceptor with Ig and ITIM domains
TAMs: tumor-associated macrophages
Tregs: regulatory T cells
MDSCs: myeloid-derived suppressor cells
SCFAs: short-chain fatty acids
HDAC: histone deacetylases
LEfSe: linear discriminant analysis effect size
AHR: aryl hydrocarbon receptor
Nrf2: NF-E2-related factor 2
GCP: glycyrrhiza polysaccharide
Lachnospiraceae_UCG_001: Lachnospiraceae unclassified group 001
Ruminococcaceae_UCG_010: Ruminococcaceae unclassified group 010
Ruminococcaceae_UCG_014: Ruminococcaceae unclassified group 014
mTOR: mehanistic target of rapamycin
LBP: Lycium barbarum polysaccharides
PMI: Phosphomannose isomerase
ABCG2: ATP-binding cassette sub-family G member 2
PI3K/AKT: Phosphatidylinositol 3-kinase/Protein kinase B
F/B: Firmicutes/Bacteroidetes
RSV: resveratrol
